# A systematic review on predicting PV system parameters using machine learning

**DOI:** 10.1016/j.heliyon.2023.e16815

**Published:** 2023-06-02

**Authors:** Md Jobayer, Md Al Hasan Shaikat, Md Naimur Rashid, Md Rakibul Hasan

**Affiliations:** BRAC University, Dhaka 1212, Bangladesh

**Keywords:** Photovoltaics, System parameter estimation, Machine learning, Systematic review

## Abstract

Due to the growing demand, assessing performance has become obligatory for photovoltaic (PV) energy harvesting systems. Performance assessment involves estimating different PV system parameters. Traditional ways, such as calculating solar radiation using satellite data and the IV characteristics approach as assessment methods, are no longer reliable enough to provide a reasonable projection of PV system parameters. Estimating system parameters using machine learning (ML) approaches has become a reliable and popular method because of its speed and accuracy. This paper systematically reviewed ML-based PV parameter estimation studies published in the last three years (2020 – 2022). Studies were analyzed using several criteria, including ML algorithm, outcome, experimental setup, sample data size, and error metric. The analysis revealed several interesting factors. The neural network was the most popular ML method (32.55%), followed by random vector functional link (13.95%) and support vector machine (9.30%). Dataset was sourced from hardware tests and computer-based simulations: 66% of the studies used data from only computer simulation, 18% used data from only hardware setup, and the 16% experiments used data from both hardware and simulations to evaluate different system parameters. The top three most commonly used error metrics were root mean square error (29.1%), mean absolute error (17.5%), and coefficient of determination (15.9%). Our systematic review will help researchers assess ML algorithms' projection in PV system parameters estimation. Consequently, scopes shall be created to establish more robust governmental frameworks, expand private financing in the PV industry, and optimize PV system parameters.

## 1 Introduction

Energy harvesting systems, such as coal-fired and nuclear power plants, are among the most polluting elements of our environment. Scientists and researchers are working hard to create a clean energy-harvesting environment. Hydroelectric power plants, wind turbines, and photovoltaics are some of the best-known alternatives to those non-renewable energy sources. In photovoltaic systems, electricity is produced utilizing the light and the heat generated from the sun. Solar energy is so common because it has no resource cost and is known as an illimitable energy source [1]. As a result, energy consumers are rapidly being adapted to solar-based power systems. For example, in 2020, almost 12.30% of total renewable energy came from solar energy in South Korea, whereas in 2012, it was only about 3.04% [2].

Due to the solar orbital motion, the sun cannot provide the same irradiance throughout the whole world at the same time [1], [3]. Sometimes it provides the peak irradiance and sometimes averages throughout the day. In our modern industrial world, producing maximum power in the shortest amount of time is crucial. To harvest maximum power by forecasting and analyzing photovoltaics (PV) performance, reliable solar cell modeling is a critical factor to consider [4]. Complex machine learning (ML) models can predict a PV system's output current-voltage (I-V) and power-voltage (P-V) parameters with very high accuracy [5]. Because of its low-cost setup and reliability, its usage in grid distribution networks is also increasing day by day. Due to these facilities, the government subsidizes and provides frameworks that ultimately accelerate the implementation and scalability of PV systems [6], [7]. However, for all of this to happen, there is always a concern about their return on investment, whether it is worth their time and effort, which ultimately necessitates predicting PV performance through system parameters.

IV curve comparison, various diode models, and thermal models are some of the traditional ways to predict the PV system parameters. However, these models are very complex to implement or need better accuracy [8]. To overcome the limitations of these traditional models, ML has become one of the best alternatives because of its speed and accuracy. Also, collecting recent works and analyzing their findings would be a matter of toil and trouble for anyone interested in working and contributing to the photovoltaic industry. Considering all these factors, we have compared and analyzed the literature on PV system performance prediction based on different ML algorithms.

Mainly there are two types of prediction for PV system characterization: direct and indirect. A direct prediction system predicts PV power by training an ML model using existing PV power data. On the other hand, indirect prediction estimates the performance depending on system parameters like solar irradiance and temperature, which is not directly related to PV power parameter. In our review paper, we considered both prediction types to keep the comparison fair and balanced. ML performance is generally quantified in terms of error metrics, such as root mean square error (RMSE), mean absolute error (MAE), and mean relative error (MRE). Apart from these metrics, while reviewing the articles, we have analyzed the performance based on several other metrics like mean absolute percentage error (MAPE) and root relative squared error (RRSE).

Our work was further motivated by the absence of a comprehensive review of the latest advancements in predicting parameters for the PV systems. The latest SLR on PV performance using ML was published in early 2021 [9]. They cited only four reference works from 2020, which is logical as they completed the review in 2020. Sobri et al. [10] conducted a comprehensive analysis of various techniques including statistical methods based on time-series data, physical models, and ensemble approaches to forecast parameters of photovoltaic (PV) systems. Their findings indicated that the utilization of artificial intelligence models had the potential to significantly reduce errors in comparison to conventional approaches. Antonanzas et al. [11] stated that the uncertainty in available solar resources challenges the reliable prediction system. In the case of economic analyses, researchers mainly focused on probabilistic prediction rather than regression analysis. Even statistical techniques were found to outperform traditional parametric techniques. Because of the convenience of the modeling process, the most recent articles in their work preferred to adopt the ML technique, which enhanced the prediction performance. Similarly, it has also been shown that convolutional neural network (CNN) performs the best in collaboration with other ML methods when they are used for short-term forecasting purposes [12]. Therefore, these works show that researchers have been using ML methods to predict PV performance for a long time. However, literature review papers on these works are either not recent or did not follow any systematic approach. We, therefore, present a comprehensive review paper on this topic for the articles published from 2020 to 2022.

We present the status of current progress in PV research in terms of ML-based algorithms, how much more efficient they are compared to the old PV parameter prediction methods, and what is left unexplored. In terms of its contribution, this paper not only saves substantial time in the search for papers on machine learning-based prediction of PV system parameters but also serves as an initial reference for individuals embarking on their exploration of PV systems.

We organized our paper as follows. Firstly, in section 2, we provided a brief discussion on the significance of predicting parameters in PV systems and its potential for enhancing overall efficiency while reducing operational costs. Also, we have discussed a few limitations of the methods other than ML that were used to predict and evaluate PV system parameters. In section 3, we have discussed the general workings of some of the most popular ML models with the help of block diagrams. We have also discussed the advantages and disadvantages of these models so that we can choose which of them is appropriate for which purposes. Our research steps and strategies are presented in section 4, with a brief introduction to review papers. We have explained the research questions and discussed why their answers are valuable in terms of state-of-the-art findings. Subsequently, we addressed the procedures employed in our search, including the criteria for determining which articles to include or exclude, the search engines utilized, and the specific queries used to identify the final set of articles. In section 5, we have presented several tables and graphs to explain our findings, which will aid future scholars in making appropriate decisions regarding various factors related to the PV systems. In section 6, we identified the study gaps and the future scope of those studies, which includes direct contributions from the authors' works and an analytical report based on all of the publications we reviewed. Finally, we concluded in section 7, commenting on the current works done on PV systems along with some of the limitations of our work.

## 2 Necessity of ML-based PV parameter prediction

A microgrid is a modern approach that combines electricity from distributed power generation sources and diverse modules for storing energy, serving local electricity demands. An energy-storing facility creates a symbiotic relationship between conventional and renewable energy sources. Because of its independent functioning in grid-connected mode, the microgrid system is favored over conventional electricity distribution approaches. The efficiency of renewable energy systems plays a vital role in the functioning of a microgrid. However, intermittent and unpredictable sources, such as PV modules, provide difficulties in demand, supply, and operation. Environmental elements like solar irradiance depend on other meteorological factors, like weather systems, which include humidity, air, and other parameters. It eventually affects the functioning of the microgrid as a whole. However, to fully leverage the benefits of decentralized electricity generation, it is crucial to maintain a balanced equilibrium between electricity generation and demand. In this case, ML models can work as reliable sources to forecast solar irradiance performance, allowing system operators to plan improved scheduling, energy distribution, proper maintenance, an uninterrupted power system, and other operational features [13].

The ESS is one of the most reliable and consistent energy sources during peak hours while saving energy during off-peak hours. Photovoltaic systems are a common source of energy for the ESS system. However, because of the volatility of these photovoltaic sources induced by the varying solar irradiation, it is critical to anticipate output power to ensure an accurate estimate of loads and power generation. Rajamand et al. [14] optimizes ESS size and location by combining ANN with MLP and GNN. It resulted in up to 31% cost savings because of PV power prediction with an optional ESS deployment. So, another important aspect of accurate PV power prediction is minimizing the microgrid system's production and distribution costs.

Several solutions existed before the advent of machine learning technologies to justify PV performance. Methods like the ideal model, the four-parameter model, the single-diode or five-parameter model, and the double-diode or seven-parameter model are examples of non-machine learning approaches [15]. These models rely on a few distinct PV module-related characteristics and a few meteorological attributes. However, in most cases, a PV module's performance and quality are determined by four factors that are influenced by the intensity of solar irradiation and the module's temperature [16].

Generally speaking, there are two ways to assess the performance of these models. The first technique is computing the instantaneous peak power under certain circumstances. The second method involved regression analysis, which used long-term data supplied by existing PV modules. The first approach is tested under standard test conditions, whereas the second is tested under utility-scale and normal cell temperature test conditions. The standard tests are commonly carried out under specific parameters, including an air mass equivalent to 1.5, a cell temperature maintained at 25 degrees Celsius, and an irradiation spectrum of 1000 $W/m^{2}$. On the other hand, the second testing method is carried out at 20 degrees Celsius under 1000 $W/m^{2}$ of illumination [17]. However, maintaining these standards in real life is quite challenging. As a result, the test findings differ and are falsified. Another frequent issue is the second test's requirement for a significant amount of data before the regression analysis [18]. Also, the limited computation capacity hampers the testing procedure. Therefore, it might result in inaccurate output due to data unavailability and a lack of processing resources.

Various models concentrate on the thermal characteristics of the PV modules in addition to these electrical equivalent models. While some models are based on thermal capacitance [19], others are based on the total heat loss coefficient [20], [19]. However, as the manufacturers do not offer adequate details about these features, these models are not realistic to use. Haouari-Merbah et al. [21] proposed a novel model that effectively captures the IV curve by delineating it into two distinct regions, facilitating the extraction of the physical parameters. But rather than offering comprehensive statistics, this model provides data on three PV parameters.

## 3 Machine learning models

### 3.1 Neural network

**Figure 1:**
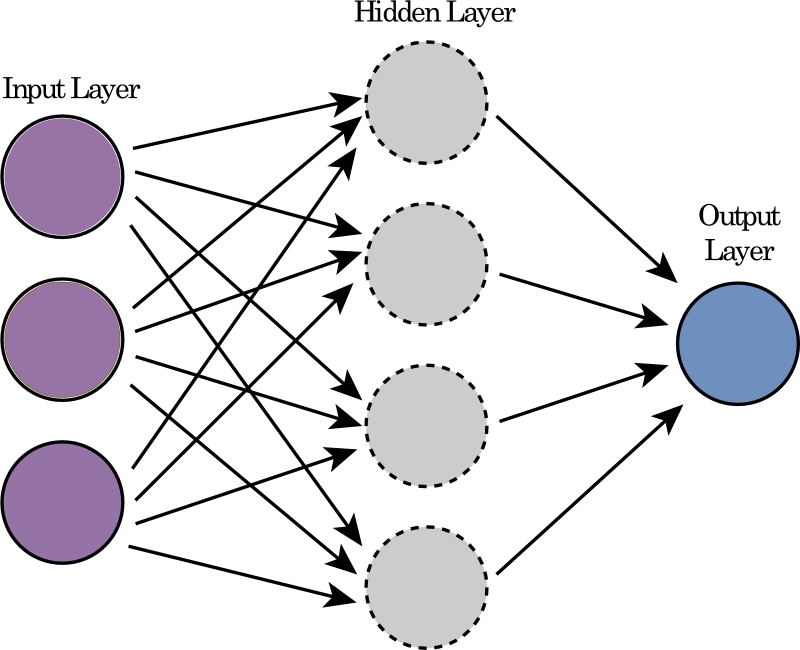
Neural Network block diagram.

A neural network (NN) replicates the functioning of neurons in the human brain and lies at the heart of pattern recognition. It consists of three essential elements: an input layer, one or more hidden layers, and an output layer (Fig. 1). These layers consist of neurons having network parameters (weights and biases) [22]. All the data is fed into the input layer. The network can incorporate one or multiple hidden layers followed by activation functions to handle and analyze the input data. Finally, the output layer generates predictions based on the input data provided.

#### 3.1.1 Advantages

NN can quickly handle problems with uncertain behavior or structure using its non-linear activation functions. It is well recognized for its adoptable mechanism; that is, it can change its structure depending on the purpose of its usage. It is possible by taking full advantage of the cognitive abilities that lie within its algorithm. The input data that pass through the network of a NN determines how it modifies its pattern. Because of its non-linear activation function, it can work with data of any dimension as long as the input is a continuously differentiable function.

#### 3.1.2 Disadvantages

The primary drawback of NN is that it demands a lot of computer resources due to the vast amount of input data requirements. To improve prediction performance, a large amount of training data is needed. Another issue is that it is susceptible to the initial randomization of network parameters. Furthermore, the rate of processing time also increases exponentially as the number of hidden layers increases.

### 3.2 Decision tree

**Figure 2:**
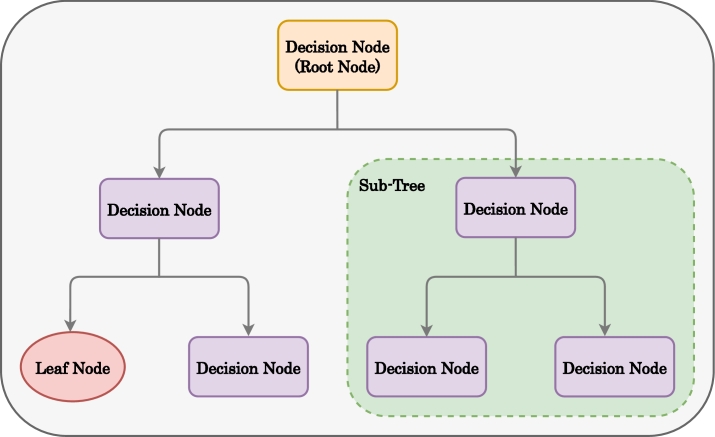
Decision Tree block diagram.

As shown in Fig. 2, a decision tree (DT) initiates by establishing a root node that branches out into multiple child nodes. These child nodes encompass both leaf nodes and decision nodes. While the root node itself is a decision node, the algorithm restricts the presence of only one root node throughout its entire operation. However, in the case of a child decision node, it can have more child nodes, including leaf nodes and other decision nodes. Leaf nodes are not divisible further. They are the output for a particular decision case. Also, the whole evaluation consists of all sorts of nodes and is called a tree. However, within a tree, it is possible to have multiple sub-trees consisting of leaf and decision nodes.

#### 3.2.1 Advantages

The first benefit of a decision tree is that it can solve both regression and classification tasks. Next, neither standardization nor normalization is necessary for it. It is built on a rule-based methodology as opposed to computing data distance. Additionally, scaling of data features is not necessary. Contrary to curve-based algorithms, the DT method is unaffected by non-linear parameters. Other benefits include: (1) non-parametric behavior, (2) excellent efficiency due to the tree traversal technique, and (3) the ability to fill in missing values with the best suitable one.

#### 3.2.2 Disadvantages

The primary issue with decision trees is overfitting. The outcome is anticipated inaccurately as a result. Sometimes, when the algorithm tries to fit the data, it keeps producing new nodes after each iteration, which makes the method harder to comprehend. Additionally, because of the volatility of the testing data, data overfitting might result in a substantial degree of inaccuracy. Furthermore, data containing many features may delay the prediction and make the system less effective.

### 3.3 Support vector machine

**Figure 3:**
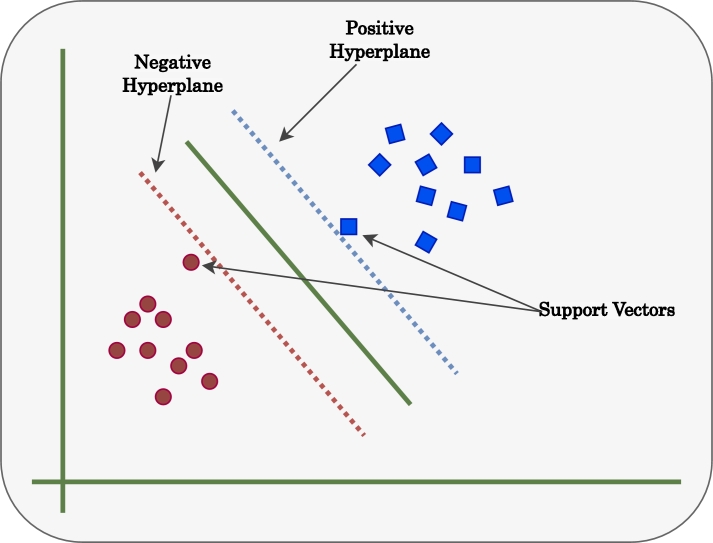
Support Vector Machine block diagram.

The support vector machine (SVM) algorithm is used to classify data based on different features available for a particular dataset. It generates a better output in the case of classification problems when most of the features are categorical. It also aids in determining the best fit line between various classes. These lines are called hyperplanes, as shown in Fig. 3. Hyperplanes are created with the vectors of a particular class located at the edge. For these reasons, these are called support vectors. The maximum margin between two hyperplanes is called the optimal hyperplane. In SVM, the target remains to maximize this optimal hyperplane. It ensures that all the classes are differentiable enough to make a reliable prediction.

#### 3.3.1 Advantages

The SVM provides several advantages. Firstly, it is capable of addressing both classification and regression tasks, allowing it to effectively handle diverse types of data, including structured and semi-structured datasets. Kernel functions are a very important aspect of SVM. These are a bunch of mathematical functions defined by different SVM algorithms. Multiple varieties of kernel functions exist, including linear, nonlinear, and sigmoid options. If the kernel function is appropriately developed, it can anticipate extremely complicated data thanks to its ability to use that function as a kernel. When the number of samples is less than the number of dimensions, it demonstrates greater efficacy when operating within high-dimensional spaces. Additionally, it has a lower chance of overfitting due to its universality. Thus, it avoids becoming entangled in local optima.

#### 3.3.2 Disadvantages

When the data collection is quite extensive, one of the disadvantages is that the performance diminishes with time. Additionally, it has inconsistent performance with noisy data. Following that, selecting a suitable kernel function is a challenging and time-consuming procedure. Unlike DT, the SVM algorithm is very complex. For this reason, for datasets with lots of features, sometimes it becomes challenging to interpret the outcome. Lastly, the performance of SVM deteriorates when the number of features surpasses the number of training instances.

### 3.4 Long short-term memory

**Figure 4:**
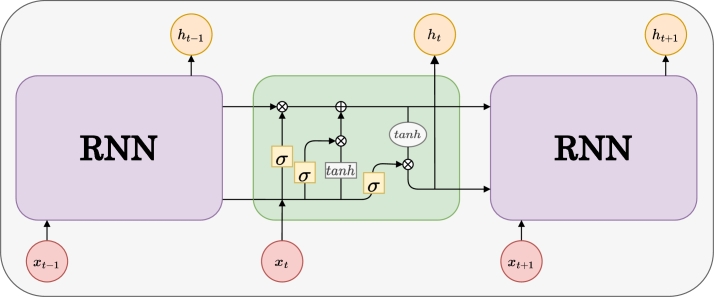
Long-Short Term Memory block diagram.

The recurrent neural network (RNN) family of models involve feedback mechanisms to process sequential data. A type of RNN is long short-term memory (LSTM) that tackles the challenge of vanishing gradients, specifically when dealing with larger datasets [23]. A RNN can predict with higher accuracy for the recently processed information. However, in the case of LSTM, it can retain information for a longer time passed through this model's memory cell. Because of its capacity to remember prior knowledge, LSTM can predict significantly quicker with higher precision [24]. The LSTM memory block is small in size but retains the memory for a more extended period. There are three gates that control the memory block: the forget gate, input gate, and output gate (Fig. 4). The forget gate is responsible for deciding which information will be retained and processed further. If it is not required, that particular information is not fed into the input gate. If it is required, the important pieces of information are fed into the input gate. In this gate, relevant information is introduced and combined with the cell state. In the output gate, the regulated data from the input gate is multiplied with the cell's tanh generated vector to extract useful information.

#### 3.4.1 Advantages

The primary benefit of LSTM is its capacity for longer-term knowledge retention. The LSTM may even filter redundant data that is no longer needed with its forget gate. It fills in the gaps left by its parent RNN model by offering extra variables, including learning rates, input biases, and output biases. The capacity to manage noise, the need for zero-fine adjustments, and distributed representations with four interacting layers are additional characteristics of LSTM.

#### 3.4.2 Disadvantages

Due to the linear layers included in its cells, the first drawback of LSTM is the need for significant computational resources to prepare it for usage in production. Similar to feedforward NN, LSTM also has problems with random weight initialization. Over time, it tends to get overfitted for a more considerable volume of data. Additionally, each of its cells becomes more complicated due to the so-called forget gates, and the model itself cannot eliminate redundant gradients between its previous and latter cells.

## 4 Methodology

A review paper represents the current progress in a particular field based on the existing literature. In broader terms, there are two types of review papers: narrative and systematic. A narrative review examines a specific scientific problem from a theoretical and conceptual standpoint comprising of critical appraisal of a state of knowledge [25], which is only sometimes evidence-based and has several limitations [26]. On the contrary, a systematic literature review (SLR) employs scientific methodologies to guarantee a methodical method for addressing a problem, conducting thorough evaluation, and amalgamating all pertinent works regarding a particular subject [27]. We incorporated a study approach adhering to the SLR standards to investigate current progress on PV system parameters estimation based on ML algorithms.

**Figure 5:**
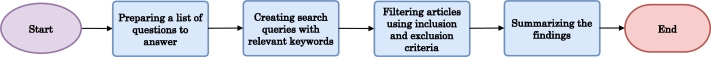
Research methodology.

Fig. 5 depicts our overall study process. We began by preparing five research questions to develop the SLR framework, which is our research's foundation. The whole research aims to answer these questions thoroughly. Next, we devised several relevant keywords and produced the appropriate search phrase, which served as our first step in shortlisting the articles. We used four inclusion/exclusion criteria to shortlist the papers further, which helped us select which ones to keep and which to discard. The following subsections go through all of the filtering techniques in depth.

### 4.1 Research questions

Questioning is the first step of every scientific investigation; SLR is no exception. We provide quantitative and analytical measures to all of our questions. Every question solves a specific issue related to the PV system parameter estimation. Below all of the questions are thoroughly explained.

**RQ1.** **Which ML methods were used in the experiment?**
  The primary item of ML-based forecasting studies is the ML algorithm itself. Therefore, by answering this question, we summarized the top-performing and some seldom exploited ML models used by different researchers.
**RQ2.** **What was the sample data size in the experiment?**
  The first aspect of appraising an experiment is knowing its data, how long it ran, and how many samples were utilized. The more data we work with, the more insights it will provide. By finding the answer to this question, we can maintain a good balance while comparing the results, as the database size heavily influences the final result.
**RQ3.** **Was it a practical or simulation-based experiment?**
  Almost every experiment has a difference between the theoretical and actual outcomes. It is much more crucial in terms of PV performance assessment since experimental outcomes are strongly influenced by external factors such as temperature, location, solar panel tilt angle, and various other parameters. In a simulation-based experiment, on the other hand, we specify some fixed values for these essential factors. As a result, these disparities must be considered when assessing the trials, and that is why we have tried to find answers to this question.
**RQ4.** **What error metrics were considered?**
  Measurement inaccuracy is an inevitable part of every experiment [28]. In the case of the PV performance test, as we mentioned previously, various extrinsic factors influence the result. Therefore, to acquire practical insights from the findings, authors may choose several error factors like MAE, mean squared error (MSE), and RMSE. Answers to these questions will help researchers to select appropriate error metrics for their experiments.
**RQ5.** **What was the percentage of errors and accuracy?**
  Quantitatively evaluating the errors and accuracies with different error metrics is very important in performance evaluation. It allows us to analyze and visualize the output as per our needs. Our article has documented all types of important quantitative findings and compared them to develop a coherent breakdown. In the following sections, we have addressed them in depth.

### 4.2 Search strategy

**Figure 6:**
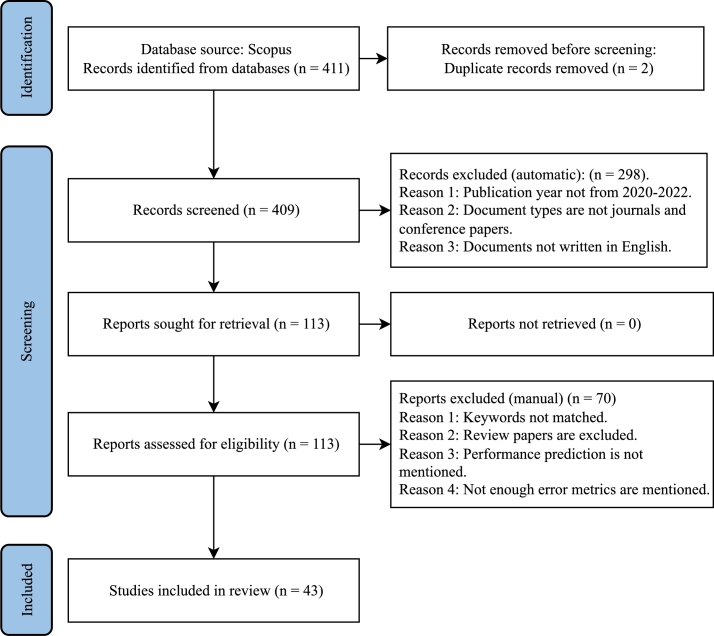
PRISMA diagram, illustrating how we selected research articles to review.

Fig. 6 illustrates our article selection process, including the database source, the number of articles initially found, the number of articles discarded automatically and manually, and the number of articles left for the final review.

#### 4.2.1 Keyword selection

We set three specific keywords: photovoltaic, performance prediction, and machine learning. The performance prediction term incorporates direct and indirect prediction based on system parameters. Different authors may use different synonyms or similar terms for these keywords. We, therefore, chose the words and phrases so that they all can be used to represent the works we reviewed in this article. Our search query comprises multiple possible synonyms and similar terms relating to the three primary keywords (Table 1).

**Table 1:** Search query used on Scopus.

TITLE-ABS-KEY((photovoltaic OR pv OR solar) AND (“performance prediction” OR “performance estimation” OR “performance forecasting” OR “performance evaluation”) AND (“machine learning” OR “artificial intelligence” OR “neural network” OR “deep learning” OR regression OR fuzzy))

We used logical terms (AND and OR) to specify relationships among the selected keywords to extract our intended article from Scopus, a database of high-quality publications. We searched the article title, abstract, and keywords using its comprehensive search facilities.

#### 4.2.2 Screening

Scopus yielded a total of 411 publications initially. We then screened them automatically using the filtering options of the Scopus search page. We chose articles published between 2020 and 2022, a period of three years. In addition, the document format was restricted to articles and conference papers. Journals and conference proceedings were chosen as source types. We further made sure that the selected papers were written in English.

After the automatic filtering, we retrieved all 113 research reports. We then manually examined and screened them according to our inclusion/exclusion criteria. Articles were excluded if they were (1) review papers, letters, or presentations, (2) focused on non-solar PV technology, and (3) did not provide any numerical data related to accuracy and error percentage. After all these stages, we finally had 43 papers to analyze in this review.

## 5 Results and discussions

### 5.1 ML methods used in the experiments

**Figure 7:**
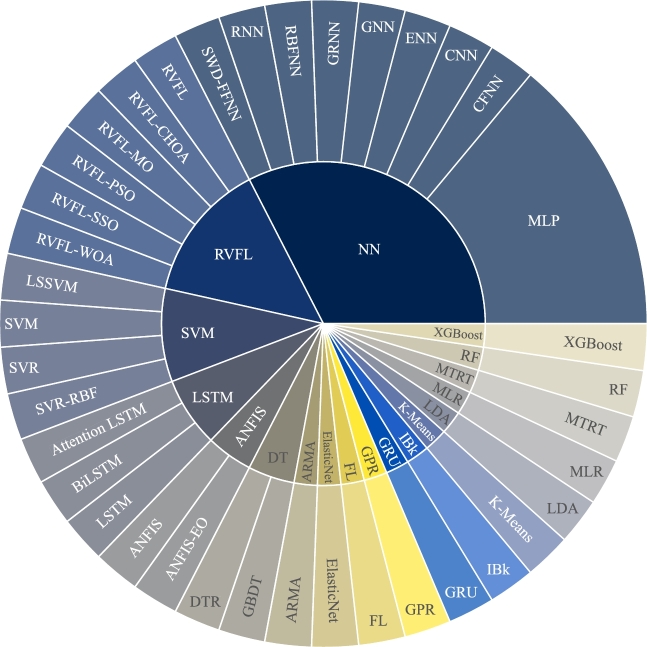
ML models usage frequency.

It is evident from Fig. 7 that the NN constitutes the largest parent group (32.55%), comprising several extensively employed methods for ML among the papers we examined. The multilayer perceptron (MLP) is used more often than the other NN methods in applications like solar system prediction. Our findings show that MLP holds the highest usage percentage among all the NN models. It is highly prevalent because of its pattern extraction capability. Unlike other ML algorithms, MLP does not need many feature extraction strategies. It automatically learns the extraction strategies from the supplied data.

The second most frequently used ML parent method is random vector functional link (RVFL) (13.95%). Unlike in MLP, where all input parameters are optimized during the training process, in RVFL, only the output weights are adapted. The rest of the parameters, like input nodes, hidden weights, and thresholds, are constrained and predicted randomly in advance. This feature minimizes the complexity of dimensionality while predicting. RVFL randomly calculates weights and biases of the input data, which is incredibly quick compared to older approaches like IV curve comparison and different diode models [29]. RVFL is the second most popular ML method because of different researchers' use of different optimizers.

As discussed earlier in section 3.3, the SVM is best known for its accurate classification capability. The PV system has many linear and non-linear characteristics. Due to these characteristics, SVM is a popular ML model for predicting these system parameters. For this reason, it occupies the third most frequently used ML parent method (9.30%), covering least squares SVM (LSSVM), support vector regression (SVR), SVR-radial basis function (SVR-RBF), and SVM itself.

As the fourth widely used ML algorithm, LSTM incorporates three subsidiary algorithms, namely attention LSTM, bidirectional (Bi) LSTM, and LSTM itself. As LSTM can retain information for longer, it has been used to predict PV system parameters. It is because the PV system usually consists of several vital parameters that must be retained for further performance prediction. Similarly, adaptive neuro-fuzzy inference system (ANFIS) and DT have two child ML models, each with almost similar popularity. The rest of the ML models and their usage frequency are shown in Fig. 7.

**Figure 8:**
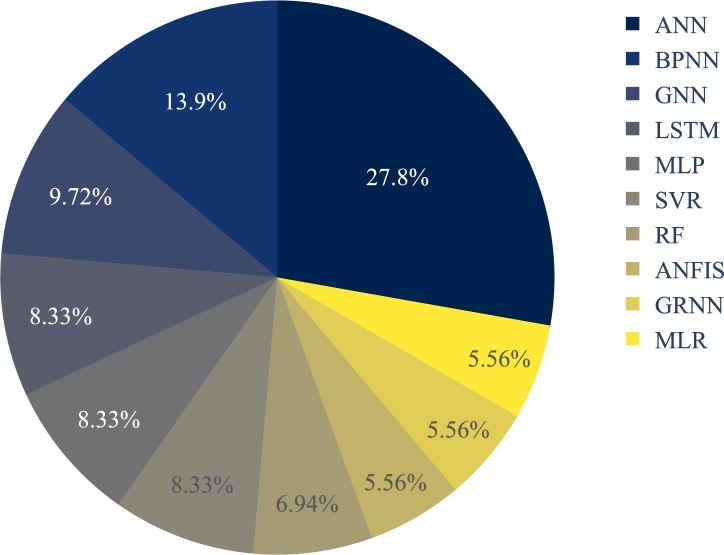
Top ten ML models.

Fig. 8 presents the top ten ML methods resulting from a narrower analysis. Fig. 7 and Fig. 8 may seem to present similar information. The former describes their usage frequency based on both the parent methods and child methods, whereas the latter depicts the usage frequency of the top ten methods regardless of their parent category.

BPNNs and GNNs are part of ANNs; the difference is whether they are trained with backpropagation algorithm (BPNN) or genetic algorithm (GNN). In Fig. 8, we followed the same naming conventions used by the respective authors and showed them separately because the difference was unclear from the articles. According to Fig. 8, artificial neural network (ANN) holds 27.8% of the use cases while evaluating the performance, followed by back propagation neural network (BPNN) with 13.9%, genetic neural network (GNN) with 9.72%, and LSTM with 8.33%. Other ML models like auto regressive moving average (ARMA) and random forest (RF) have also been used on a small scale. For example, Shahsavar et al. [30] found RF to perform better than other major ML models, including MLP and multiple linear regression (MLR).

### 5.2 Sample data size

The sample data size of the chosen publications spans from days to years, from a few hundred to several thousand samples. For example, Hashemi et al. [31] produced a massive dataset comprising more than 3.6 million data points with a rooftop installation in Denver, Colorado. However, instead of the actual number, a time interval has been presented as the sample series of measures in the more significant part of the instances. This is due to the fact that the majority of a PV panel's performance relies on the solar irradiance, and solar irradiance itself is highly dependable during daytime. For instance, Onal [1] created a PV system of dimensions 2×2 by interconnecting two panels in a series configuration and two panels in a parallel configuration, and then proceeded to simulate its performance by collecting solar irradiation data for an entire day. Along with this short-term trial, some experiments extend from one year to more than thirty years [32], [33]. In some situations, researchers have collected data at different time intervals to enhance their understanding. They have categorized data collection into long-term intervals, where observations span one day; medium-term intervals, ranging from six hours to one day; short-term intervals, spanning from thirty minutes to six hours; and ultra-short-term intervals, covering time periods from a few seconds to thirty minutes [34]. An overview of the sample data size has been presented in Table 2 to better understand the sample data size and its variations.

**Table 2:** Predicted PV parameters and sample dataset size used by different studies.

Reference	Target parameters	Sample size
[1]	Power, Irradiance	24 hours
[35]	Thermal efficiency	7 hours
[36]	Power	390
[37]	Power	5936
[38]	Thermal efficiency	12238
[39]	Power	49392
[40]	Power	24 hours
[41]	Power	-
[32]	Power	1 year
[4]	I-V characteristics	1300
[42]	Irradiance	2 years
[43]	Irradiance	43080
[44]	Energy management	40 minutes
[5]	Power	26 pairs
[45]	Power	-
[46]	Thermal efficiency	158 datasets
[31]	Power	8 years
[47]	Irradiance	72 hours
[48]	Energy yield	18
[49]	Irradiance	2 years
[50]	Power	5000
[33]	Irradiance	36 years
[51]	Power	-
[52]	Irradiance	5 - 60 minutes
[53]	Irradiance	1.2 years
[54]	Temperature, Power	1 year
[55]	Power	6 hours
[56]	Power	6 months
[30]	Power	1 year
[57]	Power	10000
[58]	Power	-
[59]	Power	10000
[60]	Power	195 pairs
[61]	Irradiance	1 year
[62]	Power	992
[63]	Fault classification	1200
[64]	Power	378 days
[65]	Power	4 days
[66]	Power	866
[34]	Power	1307
[67]	I-V characteristics	1 year
[68]	Power	169 days

*Note:* Unitless sample size refers to the number of samples.		

### 5.3 Experiment type

It is crucial to know the procedure of an experiment to evaluate its quality and other characteristics since it strongly influences the result. It is more prominent in solar experiments since researchers can postulate solar panels' performance and durability using computational tools in either simulation or practical experiments. During our screening, we found that some authors performed their studies practically in real time [35], [36], [58], and many authors adopted simulation tools with curated data sets to obtain their findings [5], [67], [4]. In another situation, simulation and practical approaches have been applied in the same experiments for a better comparative analysis [50], [61], [41].

**Figure 9:**
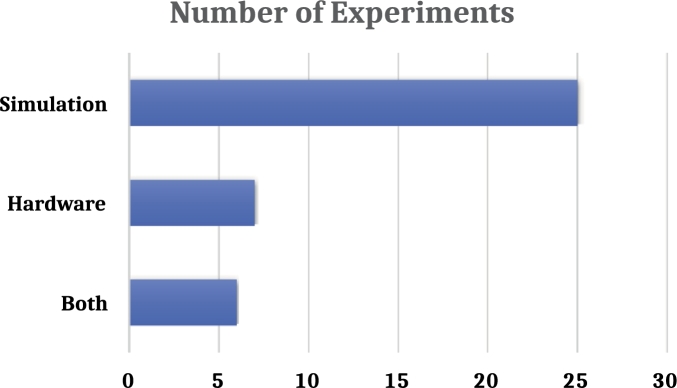
Experiment type distribution.

In Fig. 9, we observe a clear difference between the simulation and the other two (hardware and both) operations. The simulation approach has been deployed in 66% of the instances, while in the case of the hardware method, it is 18%, and in the case of both, it is 16%. One of the main reasons for simulation being the highest-picked approach might be the immediacy of the outputs. If the datasets provided in the simulation program are solid and reliable enough, it produces accurate results with minimum errors. Simulation-based studies are the most common because of their cost-effectiveness and simplicity of data manipulation. Also, several authors blended simulation and hardware methodologies to elevate the experiment's performance and dependability. This way, minimizing the experimental cost with a significant performance boost is possible.

### 5.4 Types of metrics

Among different metrics, RMSE indicates how a specific range of data has deviated from their best fit line. It corresponds to the measure of dispersion or spread exhibited by the residuals within a dataset. It is beneficial when we need to know how the predicted values have deviated from the original or the expected values. When the RMSE value is higher, it signifies a substantial deviation between the predicted values and their corresponding original values. Conversely, a lower RMSE value indicates a close alignment between the predicted and original values. In the case of PV systems, features—such as temperatures, irradiance, and power efficiency—greatly vary due to external environmental factors. RMSE and similar error metrics provide a visual representation of the deviation between the predicted performance and the actual performance. That is why RMSE was the most used error metric utilized by the authors to evaluate their works. When presenting the RMSE, it is assumed that the errors are unbiased and conform to a normal distribution. The purpose of RMSE is to offer a comprehensive depiction of the error distribution [69]. Hence, RMSE is a suitable option for evaluating PV performance.

MAE determines the precision of continuous variables and provides the mean difference between measured and actual values. Since it offers absolute values, it provides information on the size of the divergence but not its direction. A prediction's accuracy increases with decreasing MAE score. An MAE has a minimum value of zero, indicating no difference between the actual and anticipated data. Compared to the RMSE metric, this error index in the PV sector is more helpful in evaluating a number of parameters. It is because RMSE squares the error, occasionally distorting the intended meaning and introducing further problems.

The coefficient of determination ($R^{2}$) is a statistical approach for determining how well a model predicts. It is often referred to as the goodness of fit. It describes how a dependent variable responds to changes in an independent variable. $R^{2}$ can have a value as low as zero and as high as one. Values near one indicate that a model is better at predicting, whereas near zero indicates a model cannot accurately predict. This statistical technique is helpful in the context of PV systems since PV parameters, such as power efficiency and DC to AC conversion, are influenced by several independent factors. $R^{2}$ helps researchers relate those metrics to the PV systems.

**Figure 10:**
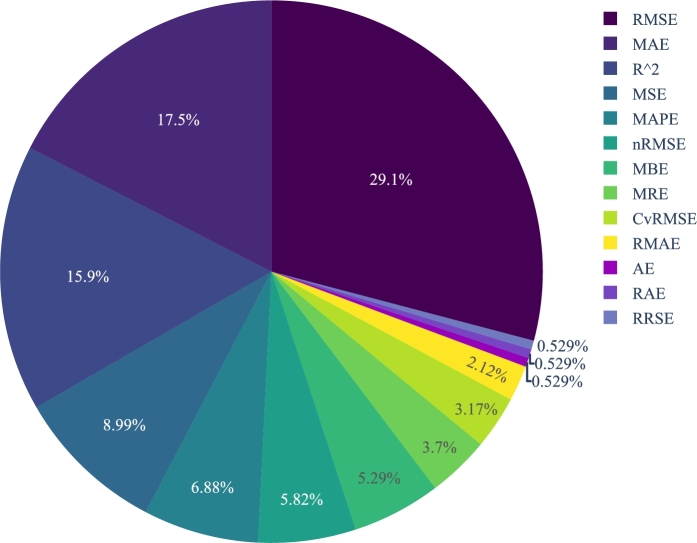
Different types of error metrics.

Fig. 10 shows the complete comparison among different error metrics based on their usage. Just 11.6% behind the most popular error metric (RMSE), the second position was secured by MAE. The third and fourth most popular error metrics are $R^{2}$ and MSE; they differ by a negligible percentage (17.5% and 15.9%, respectively). However, the fifth error metric, MAPE, differs significantly from its immediate most used error metric. The last three least used error metrics—absolute error (AE), relative absolute error (RAE), and RRSE—have the same use cases of only 0.529%.

The AE is not commonly employed as an error metric due to its limited ability to offer meaningful insights. It merely represents the absolute magnitude of the difference between the exact and measured values, without providing further informative details. RAE is another metric used by researchers to justify solar performance [30]. Solar irradiance is a complex system that provides luminance differently depending on the daytime and weather conditions. For a simple error metric like RAE, it is almost impossible to predict such a complex system, which is why it is not so popular in the case of solar performance evaluation.

### 5.5 Error percentage and accuracy

Table 3 presents that various ML methods have performed better in different experiments depending on their experimental setup, criteria, location, weather, and, most importantly, the simulation data. For instance, Onal [1] conducted a study across 12 cities located in a representative climate zone in China. Their findings revealed that the cold, hot summer, and cold winter zones were the most favorable environments for their designed system. SVR was found to be the best performing ML method in their experiments producing MSE of 0.000011548 and RMSE of 0.0034. Likewise, solar PV performs very well in hotter regions, as shown in [38], [31], [49], which is not very surprising as the higher the temperature, the higher the voltage we get from our solar cells [70]. In hotter areas, the simulation data are more stable and do not vary much, which helps predict more accurate results.

**Table 3:** Metrics based on ML methods.

Reference	Proposed methods	Best method	Metrics (Best method)
[1]	ANN, SVR, SWD-FFNN	SVR	MSE (0.000011548), RMSE (0.0034)
[35]	ANFIS-EO, ANFIS, ANN	ANFIS-EO	RMSE (0.00095), MAE (0.17140)
[36]	DL	DL	*R*^2^ (96.42%)
[37]	MLR, ElasticNet, SVR, SVR-RBF, DTR, RF, GBDT, XGBoost	XGBoost	RMSE (0.0519), MAE (0.3706)
[38]	ANN, LS-SVM	ANN	MAE (0.000078)
[40]	RVFL, RVFL-MO	RVFL-MO	*R*^2^ (99.9%), RMSE (1.63)
[41]	RVFL, RVFL (PSO, CHOA, SSO, WOA)	RVFL-CHOA	*R*^2^ (99.916%), RMSE (0.00047), MRE (0.00110), MAE (0.00167)
[32]	ANN	ANN	nRMSE (8.93%), MAPE (7.88%)
[4]	GNN (ABC, BSA, GWO, MFO, PSO, WCA, WOA)	GNN-WOA	RMSE (0.0181)
[42]	ANN, SVR, GPR	GPR	nRMSE (40.20%), DMAE (0.08912)
[43]	LSTM, CNN, CNN-LSTM, LSTM-CNN	LSTM-CNN	RMSE (0.9525), MAE (0.6675), RMAE (0.7937)
[5]	BPNN (ABC, BSA, GWO, MFO, PSO, WOA, EO)	BPNN-BSA	RMSE (0.0194)
[45]	ANN, ANFIS	ANFIS	MRE (7.25%)
[46]	RBFNN, BPNN, MLR, SVR	RBFNN	MAE (0.0043), *R*^2^ (96.58%)
[31]	ANN, SVM, GBT	GBT	RMSE (0.0346)
[47]	BPNN, RBFNN, GRNN, MLFFNN, ANN	BPNN-PSO	RMSE (0.1911), MAE (0.1000), MSE (0.0004), MAPE (0.7484%)
[50]	ANN	ANN	MSE (0.286), *R*^2^ (82.90%)
[51]	FL (Triangular, Trapezoidal, Gaussian)	FL-Triangular	MSE (0.1338)
[54]	ANN	ANN	cvRMSE (19.81%), *R*^2^ (97.00%)
[55]	ARMA, RF, MLP, SVR	ARMA	*R*^2^ (98.8%)
[30]	ANN, MLR, MLP, RBFNN, RF, lazy.IBk, RT	RF	MAE (0.4957), RMSE (0.8153), RAE (2.9586), RRSE (2.8289)
[58]	ANN	ANN	AE (1.11), RMSE (1.71)
[61]	ANN	ANN	MBE (-1.08%), CvRMSE (9.01%), IPE (23.9%)
[62]	PSO-ANN, ANN	PSO-ANN	RMSE (0.011146), *R*^2^ (99.67%)
[64]	WPD-LSTM, LSTM, RNN, GRU, MLP	WPD-LSTM	MBE (0.67%), MAPE (2.4002%), RMSE (0.2357)
[65]	GRNN(GRNN-1, GRNN-2), Fuzzy Logic	GRNN-1	Efficiency (96.22%)
[66]	ANN	ANN	*R*^2^ (83.4%), Accuracy (95.55%)
[34]	RF, GRNN, CFNN, FFNN	RF	RMSE (0.000091), MAPE (0.0028%), MBE (0.1392%)
[68]	ANN, RNN, LSTM, MLP, RBFNN, ANFIS,DNN	LSTM	-

Authors varied time duration to yield a better outcome. Theocharides et al. [32] conducted experiments using a one-year sample dataset and determined that ANN was the most suitable model for the given scenario. Duchaud et al. [55] ran the experiment during the peak hours of the day. They ran it in France in two locations (Ajaccio and Odeillo). They conducted the experiment in different intervals, namely, ultra-short-term, short-term, and long-term. First, they conducted it at varying times from 2 minutes to 1 hour (short-term), then varying times from 10 minutes to 6 hours (long-term) at maximum. The experiment was based on those two locations' global horizontal and tilted irradiance data. In their experiment, ARMA was the top performing ML algorithm among RF, MLP, SVM, and ARMA. The normalized RMSE (nRMSE) score was 0.8% in the short-term and 1.6% in the long-term scenario.

Table 3 reveals that ANN wins in terms of best performing ML methods [38], [32], [50], [54], [58], [61]. Jung et al. [54] achieved 97% $R^{2}$ score in their testing dataset using ANN, whereas Kim and Kim [36] achieved 96.42% and Zhao et al. [50] achieved 82.90% $R^{2}$ score. Along with ANN, other ML approaches, such as BPNN, ANFIS, RVFL, and RF, were extensively applied in the PV performance tests. Prediction of ANN and BPNN becomes very accurate with increasing data.

Aljanad et al. [47], Wang et al. [5] have shown different performances using different optimizers even after using the same ML algorithms. Aljanad et al. [47] have developed a solid and persistent BPNN model to predict the global solar irradiance for tropical countries like Malaysia for an extremely short time interval, showing remarkable improvement in terms of system parameter estimation. In addition, Wang et al. [5] have proposed an enhanced equilibrium optimizer that is employed in conjunction with BPNN. This optimizer demonstrates enhanced efficiency in optimization and produces fitness values that are more reasonable. They stated that their model has the capability to enhance both the precision and reliability of optimizer when it comes to estimating photovoltaic cell parameters. We have encountered several other cases where the same ML methods with different optimizers yielded different results [41], [4]. Therefore, it is evident that the same ML methods can perform differently in different optimizers, and finding the right optimizer is an essential step in extracting the best performance.

Along with the use of different optimizers, we have seen another interesting result: the order of the combinations of ML methods used in the experiments influences the results [43]. The naive forecast was the baseline in [43]'s experiment. To check the accuracy, several locations in Europe were considered. Also, a maximum of 24 hours of forecasts were recorded and then compared using different goodness of fit for a better comparison. They experimented four different models: only LSTM, only CNN, LSTM-CNN, and CNN-LSTM. Interestingly, the last two combinations showed different results. In Ulm, Germany, LSTM-CNN outperformed the other three combinations with an RMSE of 95.25, where its nearest competitor was CNN-LSTM with an RMSE of 97.35. In Almería, Spain—the sunniest location among other experimental locations—LSTM-CNN outperformed all three other ML methods in terms of MAE and root mean absolute error (RMAE) with values of 51.89 and 76.87%, respectively. Though it is evident that LSTM and CNN combinations performed around 2% better than individual models. Yet, according to the author, these minor differences matter because they enhance the reliability and robustness of the model.

In the context of grid connection systems, Bukar et al. [71] proposed a novel approach for managing energy in microgrid systems. The author's approach utilizes a technique called the grasshopper optimization algorithm to optimize the energy management process. This optimization resulted in a reduction of fuel consumption by 92.4% and carbon dioxide emission by 92.3%. Therefore, it is evident that energy loss, power optimization, and advanced system parameters prediction is getting improved with the help of different ML algorithms.

## 6 Open issues and future research directions

After evaluating the selected publications, we discovered several study gaps and future research directions. Deficiencies include inadequate data validation, lack of enough hardware-based experiments, and lack of alternative ML approaches. Also, in case of the maximum system parameters prediction, both short-term and maximum power estimations have not yet been done altogether. So, development opportunities are still available to improve the estimation accuracy of PV system parameters. For example, hybrid ML models, hardware experiments, and data variations are some solutions that can be used to solve those issues.

Research findings are sometimes influenced by the amount of data collected and the duration of the experiment. Wang et al. [4] used hourly data and advised that the study could be extended over several years to look at seasonal changes. As discussed earlier, several authors conducted their experiments in several geolocations. However, to create a generic predictor, which is not heavily influenced by location-specific data, we will need to update the models with a lot more data from around the world [36], [43]. Some authors have advised upgrading the essential network parameters and tuning the model and data training process to increase performance [4].

Most of the studies were carried out virtually using simulation tools. Nonetheless, several scholars have advocated physically evaluating the design and confirming the results [4], [72]. Some researchers have suggested that analyzing the performance of PV systems using techniques such as data mining, big data, and the Internet of Things (IoT) could yield positive outcomes. [65]. Other strategies for improving the prediction accuracy include, but are not limited to, use of meta-heuristic techniques as an alternative approach [35], hybridizing multiple ML models for optimal prediction and statistical data weighted preprocessing to reduce the training data and increase the testing data.

## 7 Conclusion

In this review paper, we investigated the patterns in PV performance evolution over the last three years, how its performance varied over time, and how they were assessed with the help of different ML algorithms. According to our findings, the way PV performance is assessed has changed significantly. Sophisticated ML algorithms outperform traditional approaches. We classified and evaluated the articles based on five research questions. Ranking the ML methods is difficult due to the differences in locations, materials, and environmental factors, such as irradiance and humidity. Overall, it was evident after the evaluation that NN was the most popular ML parent category, followed by RVFL and SVM. Within the category of individual machine learning methods, ANN emerged as the most widely used approach, followed by BPNN, GNN, and LSTM, respectively. Our work also shows that NN is meaningful for its capacity to anticipate PV performance accurately. In terms of error quantification, a large number of researchers deployed RMSE in conjunction with additional metrics, such as MAE, $R^{2}$, MSE, and MAPE. Limitations of this study include the number of research papers and the variety of source databases. Due to a lack of available ML-based PV parameters prediction works, we had to stick with a limited number of articles. Scopus, well-known for having top-notch papers, is the only database from which we sourced the articles; however, other databases might help get new insights. Despite limitations, this paper reveals the trend and open issues in PV parameter estimations, which would help future researchers further improve the parameters estimation performance.

**Acronyms**

R2: coefficient of determination
**AE**: absolute error
**ANFIS**: adaptive neuro-fuzzy inference system
**ANN**: artificial neural network
**ARMA**: auto regressive moving average
**BPNN**: back propagation neural network
**CNN**: convolutional neural network
**DT**: decision tree
**GNN**: genetic neural network
**LSSVM**: least squares SVM
**LSTM**: long short-term memory
**MAE**: mean absolute error
**MAPE**: mean absolute percentage error
**ML**: machine learning
**MLP**: multilayer perceptron
**MLR**: multiple linear regression
**MRE**: mean relative error
**MSE**: mean squared error
**NN**: neural network
**nRMSE**: normalized RMSE
**PV**: photovoltaics
**RAE**: relative absolute error
**RF**: random forest
**RMAE**: root mean absolute error
**RMSE**: root mean square error
**RNN**: recurrent neural network
**RRSE**: root relative squared error
**RVFL**: random vector functional link
**SLR**: systematic literature review
**SVM**: support vector machine
**SVR**: support vector regression
**SVR-RBF**: SVR-radial basis function

## CRediT authorship contribution statement

All authors listed have significantly contributed to the development and the writing of this article.

## Declaration of Competing Interest

The authors declare no conflict of interest.

## Data Availability

Data will be made available on request.
